# Performance evaluation and modeling of a submerged membrane bioreactor treating combined municipal and industrial wastewater using radial basis function artificial neural networks

**DOI:** 10.1186/s40201-015-0172-4

**Published:** 2015-03-13

**Authors:** Seyed Ahmad Mirbagheri, Majid Bagheri, Siamak Boudaghpour, Majid Ehteshami, Zahra Bagheri

**Affiliations:** Department of Civil Engineering, K.N. Toosi University of Technology, Vanak square, Tehran, Iran; Department and Faculty of Basic Sciences, PUK University, Kermanshah, Iran

**Keywords:** Combined wastewater, Submerged membrane bioreactor, Treatment efficiency, Artificial neural network, Radial basis function

## Abstract

Treatment process models are efficient tools to assure proper operation and better control of wastewater treatment systems. The current research was an effort to evaluate performance of a submerged membrane bioreactor (SMBR) treating combined municipal and industrial wastewater and to simulate effluent quality parameters of the SMBR using a radial basis function artificial neural network (RBFANN). The results showed that the treatment efficiencies increase and hydraulic retention time (HRT) decreases for combined wastewater compared with municipal and industrial wastewaters. The BOD, COD, ${\mathrm{NH}}_4^{+}-\mathrm{N}$ and total phosphorous (TP) removal efficiencies for combined wastewater at HRT of 7 hours were 96.9%, 96%, 96.7% and 92%, respectively. As desirable criteria for treating wastewater, the TBOD/TP ratio increased, the BOD and COD concentrations decreased to 700 and 1000 mg/L, respectively and the BOD/COD ratio was about 0.5 for combined wastewater. The training procedures of the RBFANN models were successful for all predicted components. The train and test models showed an almost perfect match between the experimental and predicted values of effluent BOD, COD, ${\mathrm{NH}}_4^{+}-\mathrm{N}$ and TP. The coefficient of determination (R^2^) values were higher than 0.98 and root mean squared error (RMSE) values did not exceed 7% for train and test models.

## Introduction

The membrane bioreactor (MBR), especially the submerged membrane bioreactor (SMBR), has been extensively investigated and applied for municipal and industrial wastewater treatment. There are more than 2200 MBR installations in operations or under construction worldwide and most of them are for municipal wastewater treatment [1,2]. Earlier studies have already shown that MBRs can be operated at much higher efficiency than of what is needed for municipal wastewater [3,4]. Treatment performances were generally good, and deterioration of the performance was not observed [5]. Rosenberger et al. [3] studied aerobic treatment of municipal wastewater in an MBR for 535 day. The pilot plant comprised an anoxic zone to enable denitrification. The hydraulic retention time (HRT) varied between 10.4 and 15.6 hours. Treatment performance was very stable and on a high level. The chemical oxygen demand (COD) was reduced by 95%. Nitrification was complete and up to 82% of the total nitrogen could be denitrified. The excellent capability of SMBRs in the treatment of municipal wastewater has decreased the HRT to the minimum possible amount compared with conventional activated sludge processes. In other words, it seems that SMBRs are over designed for the treatment of municipal wastewater.

The interest of using MBR instead of classical activated sludge system for the treatment of industrial wastewater was demonstrated [6,7]. Zhao et al. [8] used a laboratory-scale anaerobic/anoxic/oxic membrane bioreactor system to treat heavily loaded and toxic coke plant wastewater and operated for more than 500 days. Treatment performance, acute toxicity assessment, and dissolved organic characteristics of the system were investigated. When the HRT of the system was 40 hours, the removal efficiencies of COD, phenol, NH_3_ − N, total nitrogen (TN) and acute toxicity were 89.8%, 99.9%, 99.5%, 71.5% and 98.3%, respectively. A desirable treated wastewater is water that is not only low in organic or mineral components, and free from biological entities such as bacteria, pathogens, and viruses but also cost efficient and reliable [9,10]. HRT plays an important role in the removal of pollutants in activated sludge processes coupled with membranes [11]. The amount of HRT for most of industrial wastewaters is higher than 2 days. As a result, the treatment of industrial wastewater is more expensive than treatment of municipal wastewater by considering the important role of HRT in efficiency and the cost of wastewater treatment.

The components in the industrial wastewater are in huge amount; for instance, high amount of COD and biochemical oxygen demand (BOD) [12], ammonia, suspended solid or heavy metal [13] and sometimes shock loading will happen. High strength of industrial wastewater results in the low biodegradability characteristic of wastewater [14-16]. A solution used to increase the biodegradation of the slowly biodegradable compounds is the adsorbent addition in the bioreactor [17-19]. Generally, BOD/COD equal to 0.5 is considered as readily biodegradable or easily treatable [20-24]. The BOD/COD ratio for the industrial wastewaters is not equal to 0.5 and varies from 0.117 to 0.773. The idea of combined municipal and industrial wastewater is an approach to set this ratio to 0.5 and improve the other criteria for a desirable wastewater treatment such as the total BOD to total phosphorous (TBOD/TP) ratio and the influent BOD and COD concentrations.

Treatment process models are essential tools to assure proper operation and better control of wastewater treatment plants. Considerable effort has been devoted to the modeling of activated sludge processes (ASPs) since early 1970s [25]. Some deterministic models have been developed basing on the fundamental biokinetics such as activated sludge model number one (ASM1) [26,27]. Following ASM1, ASM2, ASM2d and ASM3 models were developed. The ASM2 [28] models extended the capabilities of ASM1 to involve the biological phosphorus and nitrogen removals. Whereas, ASM3 [29,30] introduced an alternative concept to the previous ASM biokinetics and aimed at simplifying the model application. Despite the availability of ASM models, the diagnosis of the process interactions and modeling of ASP in an SMBR is still difficult [30,31]. Parameter estimation and calibration of ASM models require expertise and significant effort. Moreover, calibration has to be performed for each specific treatment system. Therefore, application of ASM models to real systems can be cumbersome and problematic [25,32].

In recent years, artificial neural networks (ANNs) have been used for monitoring, controlling, classification and simulation of ASPs. ANN is a non-parametric model which utilizes interconnected mathematical nodes or neurons to form a network that can model complex functional relationships [33]. So far, different types of neural network architectures and their performances have been studied for the purpose of neuroidentification [34-37]. It includes radial basis functions (RBFs), multi-layer perceptrons (MLPs), recurrent neural networks (RNNs), and echo-state networks (ESNs). In the literature to date, a limited number of applications of ANNs have been made to SMBRs for modeling of a plant operation [31,38]. Geissler et al. [31] used an ANN model to predict the filtration performance in a submerged capillary hollow fiber membrane treating municipal wastewater. The training procedure for the ANN was conducted based upon pilot-studies with an MBR system using a novel submerged capillary module. Good correlations were found between the predicted and measured permeability using ANN. Cinar et al. [38] have also proposed an ANN model for a SMBR treating cheese whey and evaluated its performance at different sludge residence time. The results of the training procedure for effluent total phosphate, COD, ammonia, nitrate were successful. However, the results of the testing procedure for effluent total phosphate were not as good as for the effluent ammonia and nitrate, although they were better than the results of effluent COD. Up till now, there have not been any investigations on treating combined municipal and industrial wastewater by SMBRs for the purpose of optimizing HRT or performance improvement. Furthermore, no attempt has been made on the modeling of the combined municipal and industrial treatment systems.

In order to achieve the objective of this study, it was decided to employ a type of RBF, which is most commonly used in classification problems [39]. RBFs have been successfully applied for solving dynamic system problems, because they can predict the behavior directly from input/output data [40-42]. The radial basis function artificial neural network (RBFANN) was applied to model the effluent quality parameters of an SMBR treating combined municipal and industrial wastewater. The influent concentration of parameters, HRT, mixed liquor volatile suspended solids (MLVSS), total dissolved solids (TDS) and pH were inputs of the RBFANN models. Sensitivity analyses were performed to determine the effect and importance order of each input parameter on the changes of effluent concentrations.

## Material and methods

### Pilot plant configuration

**Figure 1:**
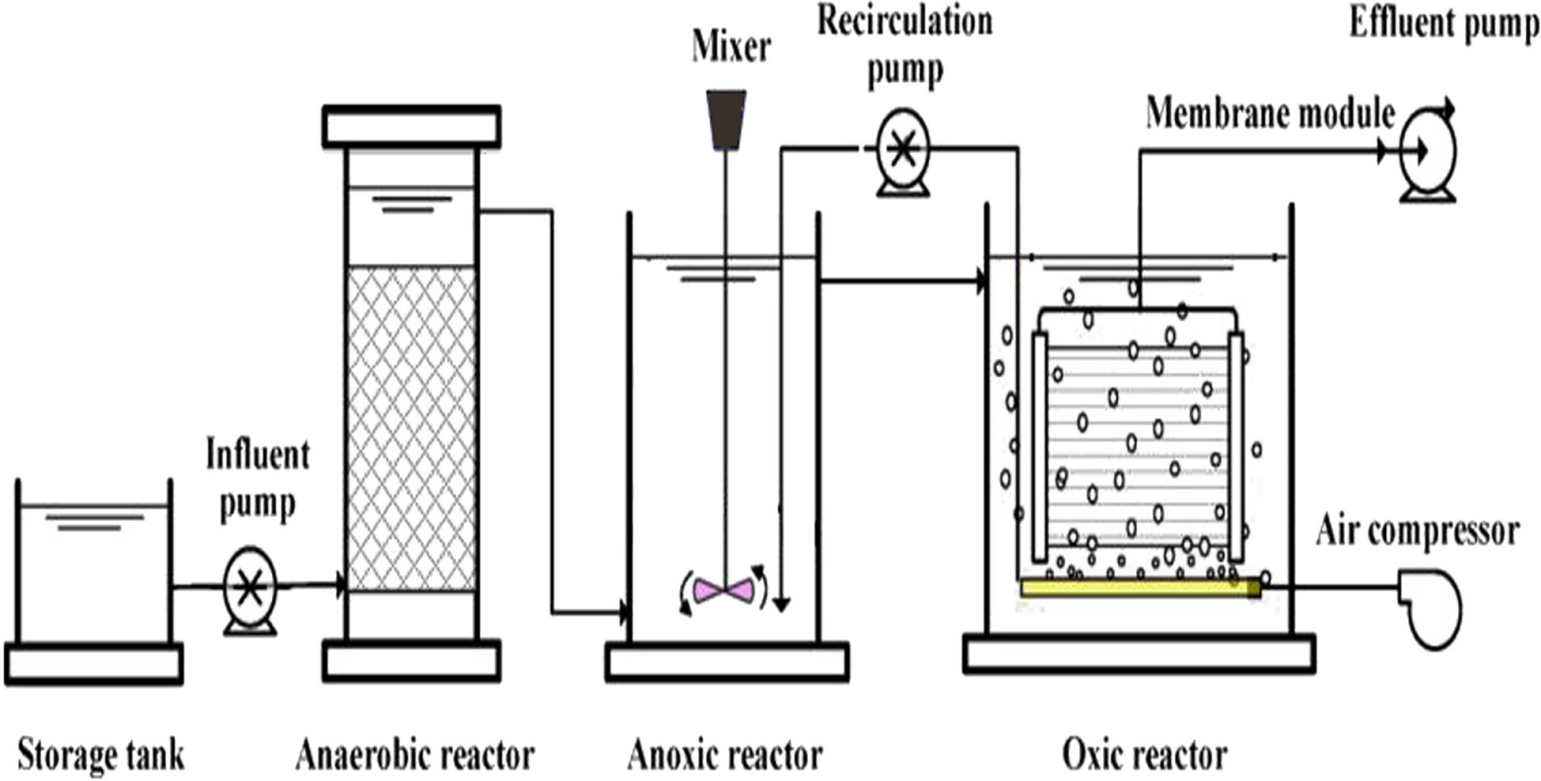
**Schematic flow diagram of the experimental apparatus.**

An SMBR was used in order to treat combined municipal and industrial wastewater in this study. Figure 1 shows the schematic diagram of the hollow fiber SMBR. The SMBR consisted of a storage tank, an anaerobic reactor, an anoxic reactor and an oxic reactor as simultaneous aeration/filtration reactor. The storage tank was made of plastic measuring 0.7 by 0.7 meter and total volume of 0.49 m^3^. The influent pump established a continuous influent wastewater flow from feeding tank to the anaerobic reactor. The anaerobic, anoxic and oxic reactors were made of Plexiglas with total volume of 0.06 m^3^, 0.1 m^3^ and 0.24 m^3^, respectively. The anaerobic reactor measuring 0.4 by 0.3 meter was located 1.2 meter above the ground level to establish a continuous flow to anoxic reactor. The anoxic reactor measuring 0.5 by 0.4 meter was located 0.9 meter above the ground level to establish a continuous flow to oxic reactor. The oxic reactor measuring 0.8 by 0.5 meter performed a simultaneous aeration/filtration treating role in the SMBR system. A small portion of the sludge in the oxic reactor was recirculated back into the anoxic reactor using a recirculation pump, where it was mixed with the effluent of anaerobic reactor. This recirculation is a key feature of the activated sludge process. The temperature was kept about 25°C and the HRT of the SMBR system varied during the experiments. The SMBR consisted of a polypropylene hollow fiber membrane with a nominal pore size of 0.04 μm. The overall membrane surface area was 8 m^2^ per module. The maximum permitted pressure for the hollow fiber membrane was about 30 kPa. A pressure gauge was installed on the suction path to turn off the suction pump and open the backwash path when the trans-membrane pressure exceeded permitted limit and membrane foiling occurred. Table 1 shows the detailed specification of hollow fiber membrane.

**Table 1:** **Specifications of the hollow fiber membrane used in this study**

**Description**	**Value**
Material	Polypropylene
Capillary Thickness	40 ~ 50 μm
Capillary Outer Diameter	450 μm
Capillary Pore Diameter	0.01 ~ 0.2 μm
Gas permeation	7.0 * 10^−2^ cm^3^/cm^2^ • S • cm Hg
Porosity	40 ~ 50%
Lengthways strength	120,000 kPa
Designed flux	6 ~ 9 L/M^2^/H
Area of membrane module	8 m^2^/module
Operating Pressure	−10 ~ −30 kPa
Flow rate	1.0 ~ 1.2 m^3^/ day

### Municipal wastewater characteristics

The pilot plant was located in Ekbatan wastewater treatment plant in Tehran, Iran, which has been operating since 1988. Influent wastewater analysis for the wastewater treatment plant was carried out for a four month period. According to the results obtained from raw wastewater analysis, the maximum values were selected as critical design parameters. Table 2 shows the critical values of the influent wastewater characteristics to Ekbatan wastewater treatment plant.

**Table 2:** **Municipal wastewater characteristics in the critical conditions**

**Parameter**	**Value**	**Parameter**	**Value**
Temperature (°C)	25.8	Org-N (mg/L)	16.8
DO (mg/L)	0	TKN (mg/L)	39.9
BOD_5_ (mg/L)	180	TS (mg/L)	810
COD (mg/L)	380	TDS (mg/L)	630
$\mathbf{N}{\mathbf{O}}_3^{-}-\mathbf{N}$ (mg/L)	0.96	TSS (mg/L)	180
$\mathbf{N}{\mathbf{H}}_4^{+}-\mathbf{N}$ (mg/L)	23.1	TP (mg/L)	16.54

### Industrial wastewater characteristics

The industrial wastewaters are defined by high strength wastewaters because of the high concentration of their components. Table 3 shows the characteristics of high strength wastewater for different industries. The COD, BOD and total suspended solids (TSS) are three most high concentration components of industrial wastewaters compared with municipal wastewater. In the current research, the high strength wastewater was simulated by increasing the influent COD, BOD, and TSS concentration to 2000, 1300 and 5000 mg/L, respectively. The simulated wastewater had the characteristics close to Beverage wastewater [12], and its TSS concentration was increased greatly. The TDS concentration was increased to 4500 mg/L and pH was different for each experiment.

**Table 3:** **Characteristics of high strength wastewater for different industries**

**Industry**	**HRT (day)**	**COD (mg/L)**	**BOD (mg/L)**	**BOD/COD**	**NH**_**4**_ **-N (mg/L)**	**TSS (mg/L)**	${\mathrm{So}}_4^{2-}$ **(mg/L)**	${\mathrm{po}}_4^{3-}$ **(mg/L)**	**Oil (mg/L)**	**Phenol (mg/L)**
Tannery [14]		2000	-	-	-	-	400	-	-	-
Tannery [12]		16000	5000	0.313	450	-	-	-	-	-
Textile [15]	2	6000	700	0.117	20	-	-	120	-	-
Textile [16]	0.7-4	4000	500	0.125	4.8	-	200	2	-	-
Dyeing [21]		1300	250	0.192	100	200	-	-	40	-
Textile [22]	0.58	1500	500	0.333	50	140	-	7	-	-
Wheat starch [10]		35000	16000	0.457	-	13300	-	-	-	-
Dairy [12]		3500	2200	0.629	120	-	-	-	-	-
Beverage [12]		1800	1000	0.556	-	-	-	-	-	-
Palm oil [23]	0.8	67000	34000	0.507	50	24000	-	-	100000	-
Pet food [24]	2.9	21000	10000	0.476	110	54000	-	200	-	-
Dairy product [18]		880	680	0.773	-	2480	-	-	-	-
Phenolic [19]	0.42	797	-	-	131	-	-	-	-	37.3
Pharmaceutical [11]	1	6300	3225	0.51	-	-	-	-	-	-

### Combined municipal and industrial wastewater characteristics

Much work has been performed to study the performance of SMBRs in the treatment of municipal and industrial wastewater, including influences of biological processes and membrane fouling. A factor which influences membrane performance in an optimum treatment of wastewater is decreasing HRT while keeping effluent components lower than discharge limits. Moreover, wastewater with BOD/COD ratio equal to 0.5 is considered as readily biodegradable. It has been shown biodegradability greater than 0.5 for spent caustic wastewater after treatment by using wet air oxidation [43]. If the ratio value is less than 0.5, the wastewater needs to have physical or chemical treatment before a biological treatment takes place [43,44]. Table 3 shows that for the most of industries this ratio is not equal to 0.5 and HRT of industrial wastewater is noticeably higher than HRT of municipal wastewater. Therefore, the municipal and industrial wastewater were combined in proportions that the BOD/COD ratio approached 0.5 for the produced combined wastewater.

### Analytical methods

Temperature, pH, dissolved oxygen (DO), BOD, COD, TSS, TDS, mixed liquor suspended solids (MLSS) and MLVSS concentration, TP, ${\mathrm{NH}}_4^{+}-\mathrm{N}$ and ${\mathrm{NO}}_3^{-}-\mathrm{N}$ were measured in this study. The pH and temperature were measured using a digital pH meter. A dissolved oxygen meter (YSI 5000) was utilized to determine DO. Biodegradability was measured by 5-day biochemical oxygen demand test according to the standard methods [45]. The seed for BOD test was obtained from the Ekbatan wastewater treatment plant [46]. The COD was determined according to the standard methods [45]. Weekly analyses included mixed liquor volatile and total suspended solids (MLVSS, MLSS) in the oxic reactor [47]. MLSS and MLVSS were determined at the Ekbatan wastewater treatment plant laboratory at the temperature of 550°C [20]. The TP and ${\mathrm{NH}}_4^{+}-\mathrm{N}$ were measured with aid of a spectrophotometer (The Hach DR 5000 UV–vis Laboratory Spectrophotometer) at the wastewater treatment plant laboratory.

### RBFANN; background and methodology

ANNs can be used for monitoring, controlling, classification and simulation of experimental data. ANNs are mathematical models simulating important parameters based on past observations in complex systems. There are many types of ANNs for modeling and function approximation of the engineering problems [35]. The two well-known ANN models, namely RBFANN and multi-layer perceptron artificial neural network (MLPANN) have been used in engineering applications to model or approximate properties [48]. An RBFANN is the most commonly used neural network for pattern recognition problems, and it is also widely used for fault diagnosis. The RBFANN has the advantages of a fast learning process, a learning stage without any iteration of updating weights, robust ability, and adaptation capability compared with other ANNs such as MLP, RNN and ESN [41,49]. RBFANNs have a very strong mathematical foundation rooted in regularization theory for solving ill-conditioned problems [48]. Tomenko et al. [50] reported that RBFANNs produced better results than multiple regression in a wetland treatment system. Madaeni et al. [51] modeled O_2_ separation from air in a hollow fiber membrane module with glassy membrane using ANN. They found RBFANN as the best network with minimum training error and checked generalization capability of their network with some unseen data (test data). Shahsavand and Pourafshari Chenar [52] found RBFANN a better choice for the prediction of gas separation performance in a membrane system because of its powerful noise filtering capability. The RBFANNs could offer more successful solutions operating on limited data (less time consuming, supplying additional information on existing relationships), thus indicating that the use of hyperspheres to divide up the pattern space into various classes is more advantageous when dealing with data described by a small number of variables [39].

The structure of the basic RBFANN consisted of one input layer, one output layer, and one hidden layer. A single-output RBFANN with N hidden layer nodes can be described by Eq. (1) and (2).

1 $$ Y={\displaystyle \sum_{n=1}^N}{w}_n{\theta}_n(X) $$

where X and Y are the input and output of the network, X = (x1, *x*2,…, xm)T, wn is the connecting weights between nth hidden node and the output layer, θn is the output value of the nth hidden node, and

2 $$ {\theta}_n(X)={e}^{\left(-x-{\mu}_n/{\sigma}_n^2\right)} $$

where μn is the center vector of nth hidden node, *x* − μ_*n*_ is the Euclidean distance between x and μn, and σn is the radius of the nth hidden node.

As its name implies, radially symmetric basis function is used as activation functions of hidden nodes [39,48]. The transformation from the input nodes to the hidden nodes is a non-linear one, and training of this portion of the network is generally accomplished by an unsupervised fashion. The training of the network parameters (weight) between the hidden and output layers occurs in a supervised fashion based on target outputs [48]. For a RBFANN, the capabilities of the final network are determined by the parameter optimization algorithms and the structure size. However, the number of hidden nodes in these RBFANNs is often assumed to be constant [53]. The transfer function of the RBFANN hidden in layer unit is symmetrical RBF (such as Gaussian function). RBF network has approximation to nonlinear continuous function, and it can learn at high speed and undertake a wide range of data fusion and high-speed processing data in parallel [54]. In this study, the RBFANN applies different network functions such as newrbe and newrb to the input data. The newrb function designs a radial basis neural network and the newrbe function designs an exact radial basis ANN.

### Model development

Simulation model of operational parameters was established based on the theory of feed forward artificial neural networks, namely RBFANN using the mathematical software program MATLAB. The operating parameters, including influent BOD, COD, ${\mathrm{NH}}_4^{+}-\mathrm{N}$ or TP as well as HRT, MLVSS, TDS and pH were the input variables of the RBFANN models. These variables were used to train RBFANN in order to simulate effluent BOD, COD, ${\mathrm{NH}}_4^{+}-\mathrm{N}$ and TP. Experimental data over 90 days (30 total data points) were used in RBFANN modeling process. Table 4 shows the statistical characteristics of the measured process variables used in this study to model effluent BOD, COD, ${\mathrm{NH}}_4^{+}-\mathrm{N}$ and TP by RBFANN.

**Table 4:** **Characteristics of measured variables used for modeling by RBFANN**

**Input variable no.**	**Input variable**	**Value**	**Output variable**	**Value**
1	Influent conc.		Effluent conc.	
	BOD (mg/L)	500–600	BOD (mg/L)	5.5–172.3
	COD (mg/L)	1000–12000	COD (mg/L)	11–396.5
	${\mathrm{NH}}_4^{+}-\mathrm{N}$ (mg/L)	21–27	${\mathrm{NH}}_4^{+}-\mathrm{N}$ (mg/L)	0.2–3.1
	TP (mg/L)	15–16.4	TP (mg/L)	1.4–6.4
2	HRT (h)	3–11		
3	MLVSS (mg/L)	4120–5990		
4	TDS (mg/L)	500–4900		
5	pH	6.2–7.6		

In order to obtain convergence within a reasonable number of cycles, the input and output data should be normalized and scaled to the range of 0–1 by Eq. (3) [55,56]:

3 $$ {x}_{ni}=\frac{x_i-{x}_{min}}{x_{max}-{x}_{min}} $$

where xi is the initial value, xmax and xmin are the maximum and minimum of the initial values, and xni is the scaled value. After the training and testing of the ANN, the output data were scaled to the real-world values through the Eq. (4).

4 $$ {x}_i={x}_{ni}\left({x}_{max}-{x}_{min}\right)+{x}_{min} $$

**Figure 2:**
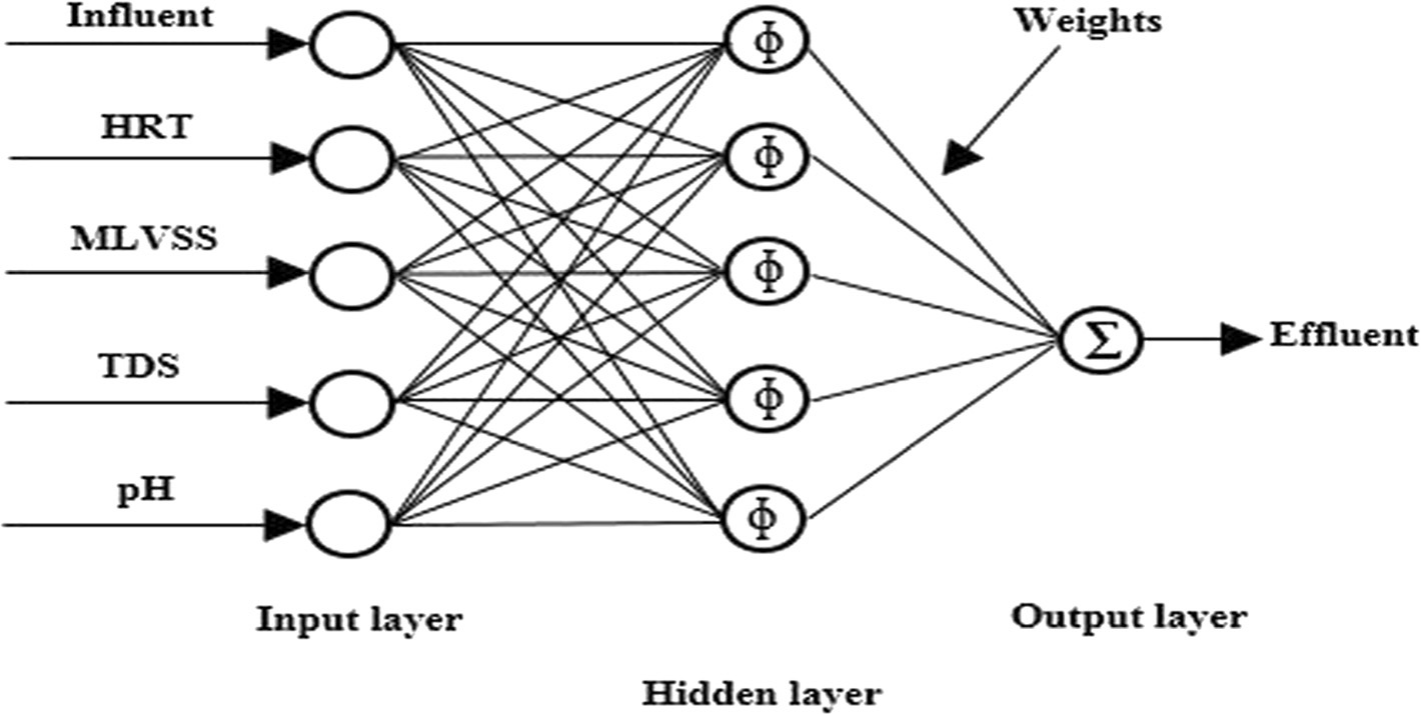
**Topological architecture of the RBF artificial neural network used in this study.**

In the current research, the developed networks consisted of three layers including one input layer that comprised five neurons (including influent BOD, COD, ${\mathrm{NH}}_4^{+}-\mathrm{N}$ or TP, HRT, MLVSS, TDS and pH), one hidden layer consisting of five neurons (which were constant due to the application of newrbe function) and the output layer that had one output neuron (which was effluent BOD or COD, ${\mathrm{NH}}_4^{+}-\mathrm{N}$ or TP). The RBFANN applied network function of newrbe (design exact radial basis network) to the input data. The newrbe function creates a two-layer network with biases for the both layers (Figure 2). The first layer has radial basis transfer function (radbas). Consequently, it calculates its weighted inputs with Euclidean distance weight function (dist) and its net input with product net input function (netprod). The second layer has linear transfer function (purelin). Consequently, it calculates its weighted inputs with Dot product weight function (dotprod) and its net input with sum net input function (netsum). For the newrbe function, the center is selected by using the fixed centers selected at random method [57]. This function can produce a network with zero error on training vectors. The stopping criterion for the newrbe function is when the outputs are exactly the matrix of target class vectors when the inputs are the matrix of input vectors [58]. The RBFANN was designed in an innovative loop that can apply newrbe function to the input data for user defined number of times in order to minimize error.

The network was trained until the network average root mean squared error (RMSE) was minimum and coefficient of determination (R^2^) approached 1. Other parameters for network were chosen as the default values of the software. The performances of the ANN models were measured by R^2^ and RMSE between the predicted values of the network and the experimental values, which were calculated by Eq. (5) and (6), respectively [59].

5 $$ {\mathrm{R}}^2=1-\frac{{\displaystyle {\sum}_{\mathrm{i}=1}^{\mathrm{n}}}{\left({\mathrm{y}}_{\mathrm{i}}^{*}-{\mathrm{y}}_{\mathrm{p}}^{\left(\mathrm{i}\right)}\right)}^2}{{\displaystyle {\sum}_{\mathrm{i}=1}^{\mathrm{n}}}{\left({\mathrm{y}}_{\mathrm{i}}^{*}-\overline{\mathrm{y}}\right)}^2} $$

6 $$ \mathrm{RMSE} = \sqrt{\frac{1}{\mathrm{n}}{\displaystyle \sum_{\mathrm{i}=1}^{\mathrm{n}}}{\left({\mathrm{y}}_{\mathrm{p}}^{\left(\mathrm{i}\right)}-{\mathrm{y}}_{\mathrm{i}}^{*}\right)}^2} $$

where $\overset{\_}{y}$ is the average of y over the n data, and ${\mathrm{y}}_{\mathrm{i}}^{*}$ and ${\mathrm{y}}_{\mathrm{p}}^{\left(\mathrm{i}\right)}$ are the *i*th target and predicted responses, respectively.

## Results and discussion

### BOD removal efficiency and modeling outcomes

The experiments were executed from HRT of 1 to 7 h in order to optimize HRT for the municipal wastewater. The influent BOD and MLVSS concentration varied from 140 to 180 mg/L and 3380 to 5470 mg/L, respectively. The removal performance of the SMBR increased from the HRT of 1 to 5 h so the effluent decreased to 5 mg/L with removal efficiency of 97% at HRT of 5 h. The effluent BOD concentration increased and removal performance decreased after 5 h due to self-degradation of microorganisms [60]. Consequently, the kinetic constants including the half saturation coefficient (K_s_), the maximum substrate utilization rate (k) and endogenous decay coefficient (K_d_) were calculated as 113 mg L^−1^, 2.05 d^−1^ and 0.036 d^−1^ different HRTs. The influent BOD concentration decreased from 175 mg/L to 25 mg/L for the effluent concentration at HRT of 1 h. It means that the effluent BOD concentration at HRT of 1 h meets discharge limits of municipal wastewater (BOD < 30 mg/L).

The HRT varied from 5 to 20 h in order to optimize HRT for the industrial wastewater. The influent BOD concentration varied from 1265 to 1360 mg/L and MLVSS concentration changed from 4350 to 7890 mg/L. The BOD removal efficiency increased from the HRT of 5 to 17 h in a trend like municipal wastewater. The effluent BOD decreased to 8.7 mg/L with removal efficiency of 99.34% at HRT of 17 h. Then efficiency decreased because of self-degradation [61]. Consequently, the kinetic constants were calculated as K_s_ equal to 163.55 mg L^−1^ and k equal to 3.56 d^−1^, K_d_ equal to 0.013 d^−1^. At HRT of 13 h the effluent BOD concentration decreased to 42.1 mg/L with removal efficiency of 96.9%. It was concluded that the HRT of 13 h cannot meet the discharge limits and the HRT of 17 h was the optimal result for the treatment of industrial wastewater. The difference between the required HRT for the treatment of municipal and industrial wastewater denotes the noticeable difference in the cost of municipal and industrial wastewater treatment. Therefore, the idea of combined municipal and industrial wastewater was followed as a key to improve the efficiency and decrease the cost of wastewater treatment.

The concentration of components of combined municipal and industrial wastewater was between municipal and industrial wastewaters. The BOD/COD ratio for the combined wastewater was about 0.5 compared with municipal and industrial wastewaters, which changed from 0.38 to 0.5 and from 0.6 to 0.7, respectively [9]. The HRT varied from 3 to 11 h in order to decrease effluent BOD concentration to discharge limits. The influent BOD and MLVSS concentration for the combined wastewater varied from 500 to 600 and from 4120 to 5990 mg/L, respectively. The influent BOD concentration decreased from 557 mg/L to 5.5 mg/L for the effluent of BOD with removal efficiency of 99% at HRT of 9 h. The removal efficiency decreased by increasing HRT from 9 to 11 h because of self-degradation [60,61]. Consequently, the kinetic constants were calculated as K_s_ equal to 177.84 mg L^−1^ and k equal to 5.29 d^−1^, K_d_ equal to 0.011 d^−1^. At HRT of 7 h for the combined wastewater, influent BOD concentration decreased from 600 mg/L to 19 mg/L for the effluent of BOD with removal efficiency of 96.9%. Therefore, the effluent BOD concentration met discharge limits at HRT of 7 h. It was observed that the performance of the SMBR increases when the influent BOD concentration is lower than 600 to 700 mg/L, which is in a good agreement with the findings of previous studies [3,24]. We concluded that by combining municipal and industrial wastewaters, the treatability of wastewater could be improved by setting BOD/COD ratio to 0.5 and reduction of wastewater strength by decreasing influent BOD concentration to lower than 700 mg/L.

**Figure 3:**
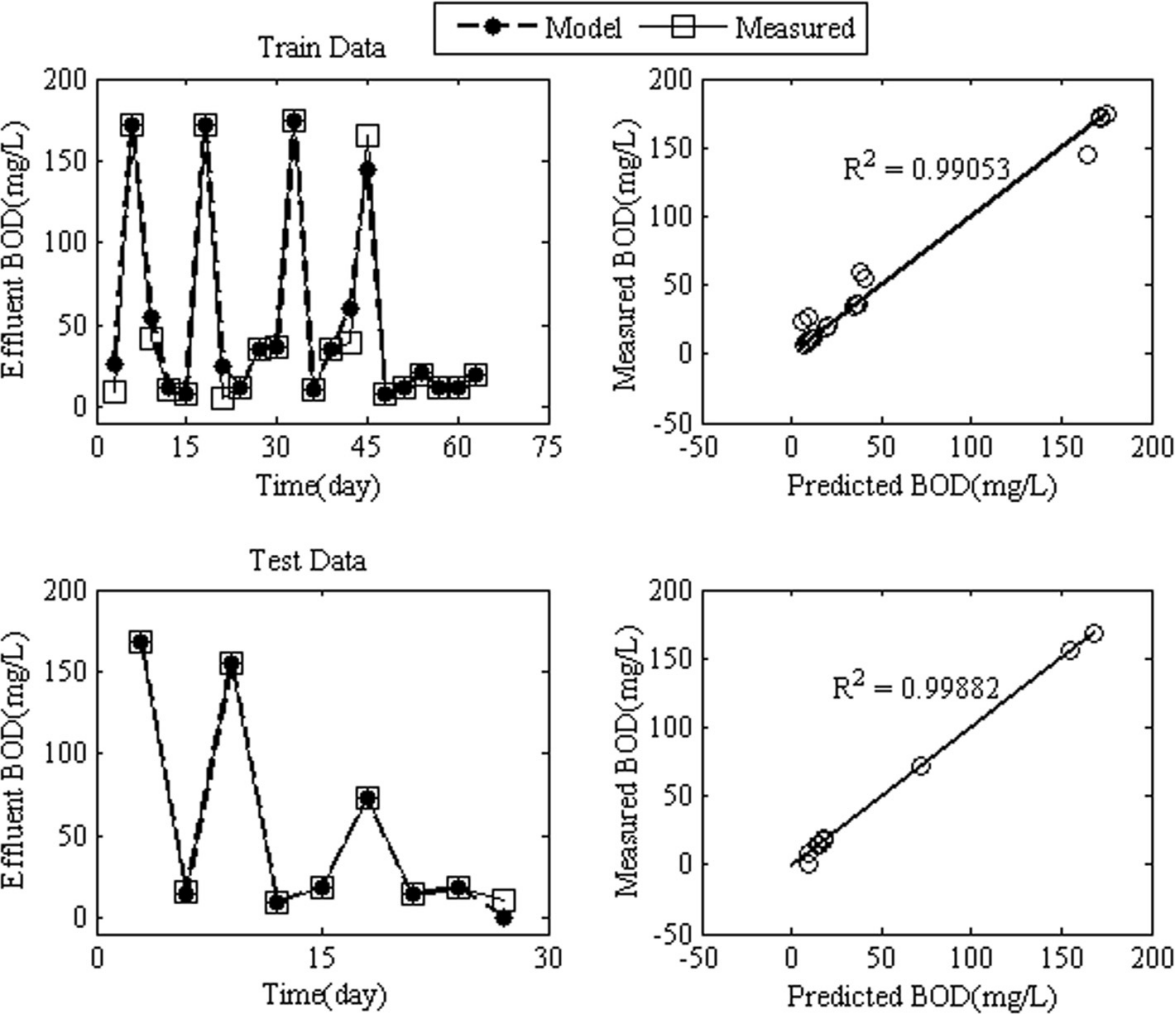
**Simulated effluent concentration of BOD by RBFANN model for train and test data.**

In order to model the effluent BOD concentration by RBFANN, the influent BOD, HRT, MLVSS, TDS and pH were input variables of the neural network. The RBFANN applied network function of newrbe to the input data and the spread of RBF was considered equal to its default value, 1. A large spread results in a smooth function approximation, but, by contrast, a large spread can cause numerical problems [48]. The network function of newrbe selected 70% of normalized data to train and 30% to test RBFANN models [38]. The RBFANN was designed in an innovative loop that applied newrbe to the data for more than 30 times in order to minimize error. Optimal network was chosen on the basis of the minimum average error. Figure 3 shows the denormalized results of the effluent BOD modeling using the RBFANN according to training and testing data sets. The results of the training procedure by RBFANN were successful for the effluent BOD concentration. Figure 3 shows that the train and test models by RBFANN indicated an almost perfect match between the experimental and the predicted effluent BOD values compared with the results of models introduced by Cinar et al. [38]. The RMSE values for train and test (verification process) models were 8.67 and 4.56 mg/L, respectively, and the R^2^ values were greater than 0.99 for both models. The RBFANN predicted effluent BOD so accurate that the mean average error for train and test models did not exceed 5% and 3%, respectively.

The effluent BOD was modeled individually by considering different single and joint variables as inputs of neural network to examine the effect of each variable on the changes of effluent BOD concentration. The joint inputs to train the neural network were groups of two, three and four variables. Table 5 shows HRT among single input variables, and HRT and MLVSS among groups of two variables had the most considerable effect on the effluent BOD concentration. Furthermore, HRT, MLVSS and TDS among groups of three variables, and HRT, MLVSS, TDS and influent BOD between groups of four variables were determined to have the greatest effects on the effluent BOD concentration.

**Table 5:** **Effect of different single and joint variables on the effluent BOD models**

**Input variable no.**	**R** ^**2**^		**RMSE (mg/L)**		**Importance order**
	**Train**	**Test**	**Train**	**Test**	
1	0.804	0.863	35.62	32.80	5
2	0.991	0.999	8.15	2.79	1
3	0.907	0.995	24.76	6.43	2
4	0.705	0.674	48.71	42.27	4
5	0.804	0.863	35.62	32.8	3
2-1	0.973	0.961	17.39	16.28	4
2-3	0.998	0.999	2.98	2.15	1
2-4	0.996	0.978	6.82	2.46	3
2-5	0.995	0.999	6.65	1.76	2
2-3-1	0.992	0.999	6.17	2.41	2
2-3-4	0.998	1	4.08	0.12	1
2-3-5	0.996	0.998	5.62	4.23	3
2-3-4-1	0.998	0.998	3.69	3.07	1
2-3-4-5	0.997	0999	4.29	3.25	2
2-3-4-1-5	0.990	0.998	8.67	4.56	1

The numbers 1 to 5 refers to input variables identified in Table 4.

The sensitivity [62] of effluent BOD concentration to changes of an input variable such as HRT determines the influence and importance of HRT on the effluent BOD models. The effect of each variable on the RBFANN models to simulate effluent of BOD compared with other variables was determined by its importance order. Table 5 shows the importance order of each input variable and the joint variables for effluent BOD. The variable with higher rank of importance denoted not only a favorable match between experimental and simulated values by RBFANN models but also low RMSE and high R^2^ values. The variation of effluent BOD concentration was influenced by HRT, MLVSS, TDS, influent BOD concentration and pH, respectively. The results of this study show that the HRT and MLVSS have the greatest influence on the effluent BOD, which are in a good agreement with earlier experimental studies [63,64].

### COD removal efficiency and modeling outcomes

To perform COD experiments for the municipal wastewater the influent COD concentration varied from 310 to 360 mg/L. By increasing HRT from 1 to 5 h the removal efficiencies increased and the best results obtained at HRT of 5 h. At this point, the effluent COD concentration reached to 8 mg/L with removal efficiency of 97.9%. The effluent COD concentration increased and removal efficiency decreased after 5 h [60]. Consequently, the kinetic constants were calculated as K_s_ equal to 96 mg L^−1^, k equal to 2.31 d^−1^ and K_d_ equal to 0.043 d^−1^. The influent COD concentration decreased from 354 mg/L to 53 mg/L for the effluent COD at HRT of 1 h. It means that the effluent COD concentration at HRT of 1 h meets discharge limits of the municipal wastewater (COD < 60 mg/L).

For the industrial wastewater the influent COD concentration varied from 2050 to 2120 mg/L. From the HRT of 5 to 17 h the removal efficiency increased and the best results obtained at HRT of 17 h. At this point, the influent COD concentration decreased from 2100 mg/L to 14.8 mg/L for the effluent COD. Consequently, the kinetic constants were calculated as K_s_ equal to 308 mg L^−1^, k equal to 2.81 d^−1^, K_d_ equal to 0.019 d^−1^. The influent COD concentration decreased from 2055 mg/L to 71.2 mg/L for the effluent with removal efficiency of 96.5% at HRT of 13 h. Therefore, it was concluded that the HRT of 13 h cannot meet the discharge limits and HRT of 17 h was considered as the optimal result.

The COD experiments were performed for the combined municipal/industrial wastewater with the HRT varying from 3 to 11 h. The influent COD concentration for the combined wastewater varied from 1000 to 1200 mg/L. The influent COD concentration at HRT of 9 h decreased from 1130 mg/L to 10.3 mg/L for the effluent of COD with removal efficiency of 99.1%. By increasing HRT from 9 to 11 h the removal efficiency decreased [60,61]. Consequently, the kinetic constants were calculated as K_s_ equal to 113.2 mg L^−1^ k equal to 2.72 d^−1^, K_d_ equal to 0.022 d^−1^. The results indicated that at HRT of 7 h the influent COD concentration decreases from 1080 mg/L to 50 mg/L for the effluent with removal efficiency of 96%. Therefore, the effluent COD concentration at HRT of 7 h met discharge limits. It was observed that the performance of the SMBR increases due to reduction of wastewater strength [9,24] when the influent COD concentration is lower than 1000 to 1100 mg/L.

**Figure 4:**
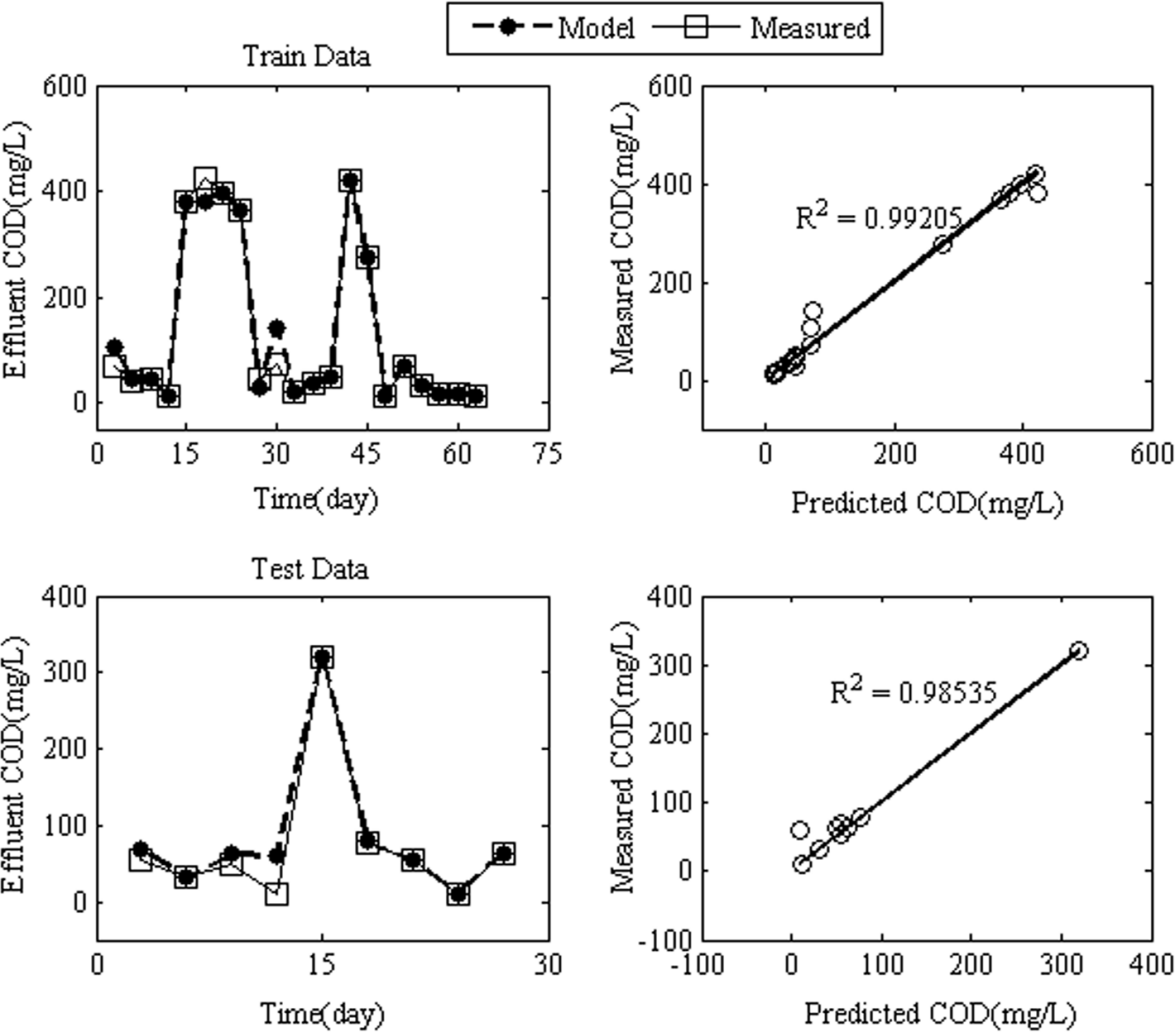
**Simulated effluent concentration of COD by RBFANN model for train and test data.**

In order to model the effluent COD by RBFANN, the influent COD, HRT, MLVSS, TDS and pH were the input variables of the neural network. The results of the effluent COD modeling using the RBFANN for train and test data were denormalized to compare the observed values of effluent COD concentration with simulated values. The results of the training procedure by RBFANN were successful for the effluent COD concentration. Figure 4 shows the train and test models by RBFANN indicated an almost perfect match between the experimental and the simulated effluent COD values compared with the results of models introduced by Cinar et al. [38]. The RMSE values for train and test (verification process) models were 25.62 and 9.12 mg/L, respectively. The values of R^2^ for train and test models were 0.99 and 0.98, respectively. The RBF models predicted effluent COD concentration with a mean average error for train and test models, which did not exceed 7% and 3%, respectively.

Table 6 shows HRT among single input variables, and HRT and MLVSS among groups of two variables had the most considerable effects on the effluent COD. Furthermore, HRT, MLVSS and TDS among groups of three variables, and HRT, MLVSS, TDS and influent COD between groups of four variables were determined to have the greatest effect on the effluent COD. Table 6 also shows the importance order of each input and joint variable for effluent COD according to sensitivity analysis procedure. The variation of effluent COD concentration was influenced by HRT, MLVSS, TDS, influent COD concentration and pH, respectively. The results showed that the HRT and MLVSS have the greatest influence on the effluent BOD, which are in a good agreement with earlier experimental studies [63,64]. The results of sensitivity analyses were highly collaborated for both BOD and COD models performed by RBFANN. The variation of both effluent BOD and COD concentration to all input variables were the same in their simulation processes, which can be justified by similarities of their natures. As a result, to control the changes of effluent BOD and COD concentration the most effective variables are HRT and MLVSS.

**Table 6:** **Effect of different single and joint variables on the effluent COD models**

**Input variable no.**	**R** ^**2**^		**RMSE (mg/L)**		**Importance order**
	**Train**	**Test**	**Train**	**Test**	
1	0.179	0.599	153.49	91.66	5
2	0.947	0.997	44.36	14.1	1
3	0.941	0.994	48.23	35.67	2
4	0.6	0.8	123	63.41	3
5	0.862	0.715	70	109.03	4
2-1	0.951	0.999	47	4.3	4
2-3	0.995	0.999	15.43	6.19	1
2-4	0.998	0.994	11.23	13.58	3
2-5	0.987	0.998	22.45	7.66	2
2-3-1	0.984	0.996	26.66	13.34	3
2-3-4	0.994	0.996	16.48	8.48	1
2-3-5	0.974	0.997	33.13	11.56	2
2-3-4-1	0.992	0.999	19.58	2.61	1
2-3-4-5	0.987	0.991	22.08	14.93	2
2-3-4-1-5	0.992	0.985	25.62	9.12	1

The numbers 1 to 5 refers to input variables identified in Table 4.

### $\mathbf{N}{\mathbf{H}}_4^{+}-\mathbf{N}$**removal efficiency and modeling outcomes**

The influent ${\mathrm{NH}}_4^{+}-\mathrm{N}$ concentration for the municipal wastewater varied from 18 to 24 mg/L. The results showed that the removal efficiency of ${\mathrm{NH}}_4^{+}-\mathrm{N}$ is improved with increase of the HRT. The influent ${\mathrm{NH}}_4^{+}-\mathrm{N}$ concentration decreased from 24 mg/L to 0.4 mg/L for the effluent at HRT of 7 h. The influent ${\mathrm{NH}}_4^{+}-\mathrm{N}$ concentration decreased from 23 mg/L to 0.8 mg/L at HRT of 5 h. As the optimal result, the effluent ${\mathrm{NH}}_4^{+}-\mathrm{N}$ concentration with removal efficiency of 96.5% at HRT of 5 h met the discharge limit (${\mathrm{NH}}_4^{+}-\mathrm{N}$ <1 mg/L). Increasing the cell retention time increases Azotobacters and Organotrophic bacteria and then followed by rapid removal of dissolved carbonaceous biochemical oxygen demand (CBOD) [20]. Rapid removal of dissolved CBOD increases the aeration time for the nitrification process [5,20]. Increases of the Azotobacter populations in activated sludge process occurs at relatively high hydraulic retention time [5,64]. Hence, the required HRT of municipal wastewater treatment was increased to 5 h.

For the industrial wastewater the influent ${\mathrm{NH}}_4^{+}-\mathrm{N}$ concentration varied from 24 to 31 mg/L. The ${\mathrm{NH}}_4^{+}-\mathrm{N}$ concentration decreased with increase of HRT for the industrial wastewater. And at HRT of 17 h the effluent ${\mathrm{NH}}_4^{+}-\mathrm{N}$ concentration was equal to 0.2 mg/L with the removal efficiency of 99.35%. The effluent ${\mathrm{NH}}_4^{+}-\mathrm{N}$ concentration at HRT of 13 h decreased from 30 mg/L to 0.7 mg/L for the effluent with the removal efficiency of 97.7%.

For the combined municipal and industrial wastewater the influent ${\mathrm{NH}}_4^{+}-\mathrm{N}$ concentration varied from 21 to 27 mg/L. The effluent concentration at HRT of 7 h met the discharge limits. The influent concentration decreased from 24 mg/L to 0.8 mg/L for the effluent with removal efficiency of 96.7%. The results of ${\mathrm{NH}}_4^{+}-\mathrm{N}$ experiments for three systems showed that the required HRT for ${\mathrm{NH}}_4^{+}-\mathrm{N}$ removal increases the required HRT for the treatment of municipal wastewater and does not have any effect on the required HRT for industrial and combined municipal and industrial wastewaters. The idea of combined wastewater is more useful because the treatment system is able to receive sewage with higher influent BOD and COD concentration without any increase in the HRT.

**Figure 5:**
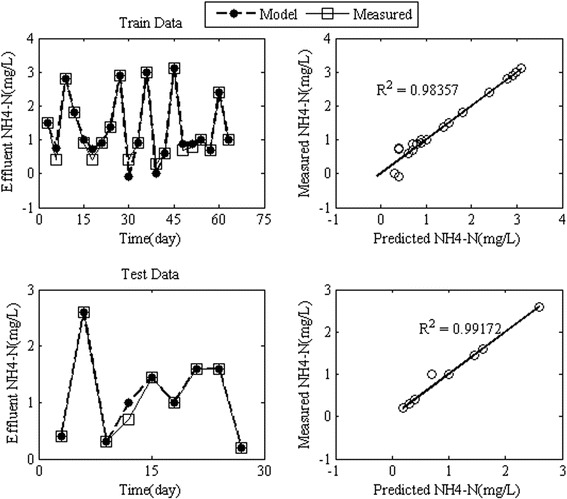
**Simulated effluent concentration of** $\mathbf{N}{\mathbf{H}}_4^{+}-\mathbf{N}$ **by RBFANN model for train and test data.**

In order to model the effluent ${\mathrm{NH}}_4^{+}-\mathrm{N}$ by RBFANN, the influent ${\mathrm{NH}}_4^{+}-\mathrm{N}$ concentration, HRT, MLVSS, TDS and pH were the input variables of network. The results of the effluent ${\mathrm{NH}}_4^{+}-\mathrm{N}$ modeling using the RBFANN for train and test data sets were denormalized to compare the observed values of effluent ${\mathrm{NH}}_4^{+}-\mathrm{N}$ concentration with simulated values. The results of the training procedure by RBFANN were successful for the effluent ${\mathrm{NH}}_4^{+}-\mathrm{N}$. Figure 5 shows that the train and test models by RBFANN indicated an almost perfect match between the experimental and the simulated values of effluent ${\mathrm{NH}}_4^{+}-\mathrm{N}$ concentration compared with the results of models introduced by Cinar et al. [38]. The RMSE values for train and test (verification process) models were 0.06 and 0.14 mg/L, respectively. The R^2^ values for train and test models were 0.98 and 0.99, respectively. The RBF models simulated effluent ${\mathrm{NH}}_4^{+}-\mathrm{N}$ with a mean average error for train and test models, which did not exceed 2% and 5%, respectively.

Table 7 shows HRT among single input variables, and HRT and MLVSS among groups of two variables had the most considerable effect on the COD effluent. Furthermore, HRT, MLVSS and pH among groups of three variables, and HRT, MLVSS, pH and influent ${\mathrm{NH}}_4^{+}-\mathrm{N}$ between groups of four variables were determined to have the greatest effect on the COD effluent. Table 7 also shows the importance order of each input variable and the joint variables for effluent ${\mathrm{NH}}_4^{+}-\mathrm{N}$ according to sensitivity analysis procedure. The variation of effluent ${\mathrm{NH}}_4^{+}-\mathrm{N}$ concentration was influenced by HRT, MLVSS, pH, influent ${\mathrm{NH}}_4^{+}-\mathrm{N}$ and TDS, respectively. The current research shows that the HRT and MLVSS have the greatest influence on the changes of effluent ${\mathrm{NH}}_4^{+}-\mathrm{N}$, which are in a good agreement with earlier experimental studies [63,64]. The results of sensitivity analysis for the effluent ${\mathrm{NH}}_4^{+}-\mathrm{N}$ models indicated that to control the changes of effluent ${\mathrm{NH}}_4^{+}-\mathrm{N}$ concentration the most effective variables are HRT and MLVSS.

**Table 7:** **Effect of different single and joint variables on the effluent** $\mathbf{N}{\mathbf{H}}_4^{+}-\mathbf{N}$ **models**

**Input variable no.**	**R** ^**2**^		**RMSE (mg/L)**		**Importance order**
	**Train**	**Test**	**Train**	**Test**	
1	0.59	0.44	0.78	0.62	5
2	0.983	0.998	0.16	0.12	1
3	0.923	0.985	0.35	0.17	2
4	0.54	0.6	0.81	0.52	4
5	0.78	0.94	0.56	0.29	3
2-1	0.99	0.993	0.13	0.13	3
2-3	0.99	0.994	0.13	0.09	1
2-4	0.99	0.964	0.12	0.23	4
2-5	0.99	0.992	0.14	0.11	2
2-3-1	0.987	0.995	0.15	0.09	3
2-3-4	0.99	0.996	0.13	0.05	2
2-3-5	0.988	1	0.14	0	1
2-3-5-1	0.99	0.998	0.14	0.06	1
2-3-5-4	0.97	0.996	0.2	0.1	2
2-3-5-1-4	0.98	0.991	0.06	0.14	1

The numbers 1 to 5 refers to input variables identified in Table 4.

### Results of TP removal efficiency and modeling

The influent TP concentration for the municipal wastewater varied from 13.2 to 15.1 mg/L. The results showed that the removal efficiency of TP is improved with increase of the HRT from 1 to 5 h. Then, the removal efficiency decreased for the HRT of 5 to 7 h. The influent TP concentration decreased from 14.8 to 2 mg/L for the effluent at HRT of 5 h with removal efficiency of 86.5%. The influent TP concentration decreased from 14.9 to 5.8 mg/L for the effluent at HRT of 1 h and met discharge limit (effluent TP <6 mg/L). The effluent TP concentration depends on the TBOD/TP ratio so with a ratio less than 20, it is not possible to achieve the effluent TP concentration lower than 2 mg/L [65]. We observed that the TBOD/TP ratio for the influent of Ekbatan wastewater treatment plant was less than 20. Subsequently, the combination of municipal and industrial wastewater was an effective method to correct this problem.

The influent TP concentration for the combined wastewater varied from 15 to 16.4 mg/L. The influent TP concentration decreased from 16.2 mg/L to 5.7 mg/L for the effluent TP at HRT of 1 h. With increasing the HRT from 7 to 9 h the effluent TP concentration reached to lower than 1 mg/L with removal efficiency of 92%. The influent TP varied from 17.24 to 20.3 mg/L for the industrial wastewater. The high ratios of the TBOD/TP for the industrial wastewater allowed the effluent TP to be lower than 1 mg/L. The effluent TP concentration reached to 0.7 mg/L with the removal efficiency of 96.55% at HRT of 17 h.

**Figure 6:**
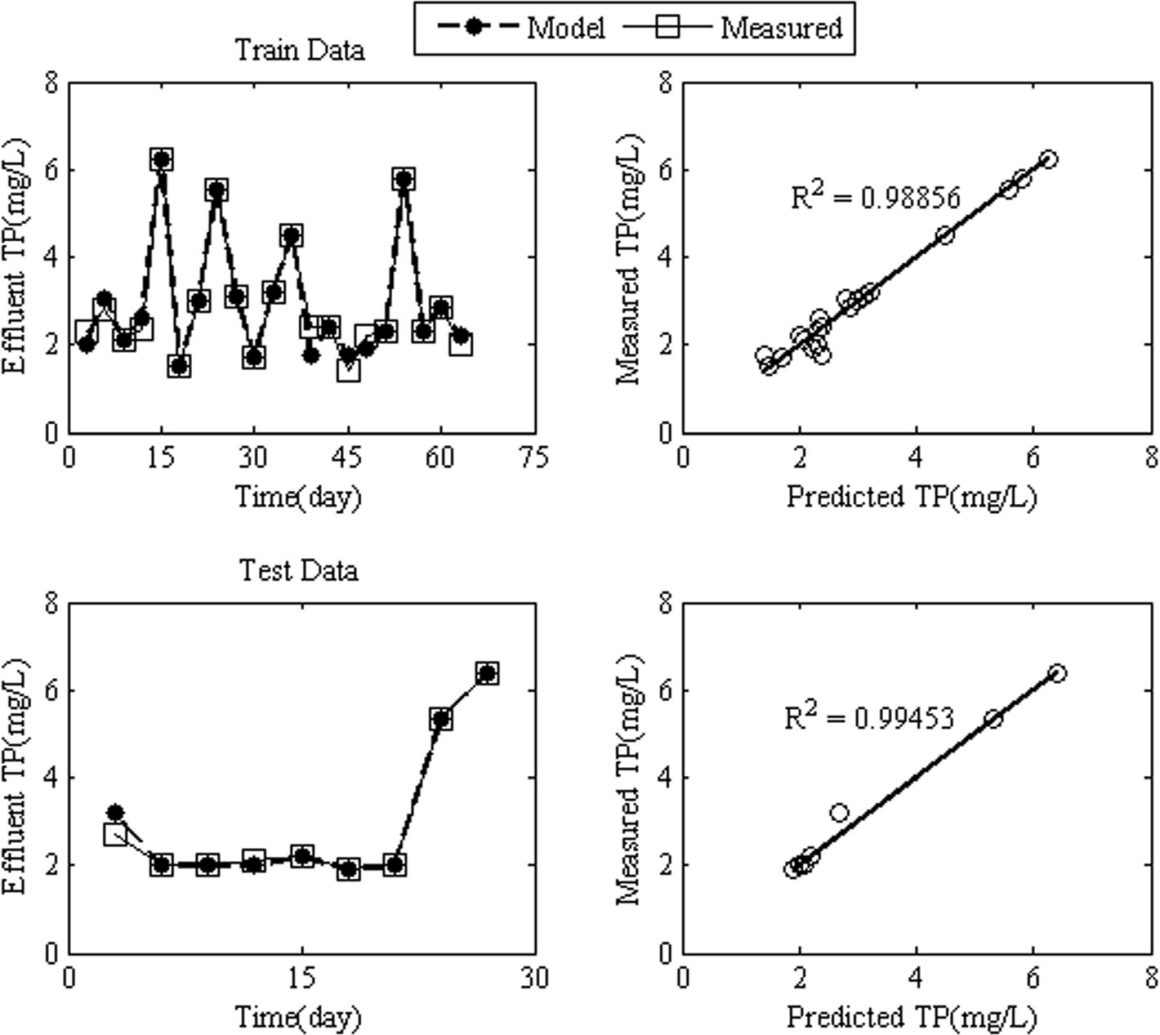
**Simulated effluent concentration of TP by RBFANN model for train and test data.**

In order to model the effluent TP by RBFANN, the influent TP concentration, HRT, MLVSS, TDS and pH were the input variables of the neural network. The results of the effluent TP modeling using the RBFANN for train and test data sets were denormalized to compare the observed values of effluent TP with simulated values. The results of the training procedure by RBFANN were successful for the effluent TP. Figure 6 shows that the train and test models by RBFANN indicated an almost perfect match between the experimental and the simulated effluent TP concentration compared with the results of models introduced by Cinar et al. [38]. The RMSE values for train and test (verification process) models were 0.32 and 0.17 mg/L respectively, and the value of R^2^ was 0.99 for both models. The RBFANN models simulated effluent TP so accurate that the mean average error for train and test models did not exceed 5% and 3%, respectively.

Table 8 shows HRT among single input variables, and HRT and pH among groups of two variables had the most considerable effect on the effluent TP. Furthermore, HRT, pH and MLVSS among groups of three variables, and HRT, pH, MLVSS and influent TP concentration between groups of four variables were determined to have the greatest effect on the effluent TP. Table 8 also shows the importance order of each input variable and the joint variables for effluent TP according to sensitivity analysis procedure. The variation of effluent TP concentration was influenced by HRT, pH, MLVSS, influent TP concentration and TDS, respectively, which is in a good agreement with earlier experimental studies [63,64].

**Table 8:** **Effect of different single and joint variables on the effluent TP models**

**Input variable no.**	**R** ^**2**^		**RMSE (mg/L)**		**Importance order**
	**Train**	**Test**	**Train**	**Test**	
1	0.34	0.3	1.15	1.88	5
2	0.972	0.992	0.35	0.14	1
3	0.878	0.978	0.7	0.29	2
4	0.51	0.8	1.39	0.82	4
5	0.77	0.96	0.99	0.4	3
2-1	0.975	0.97	0.37	0.37	4
2-3	0.99	0.99	0.22	0.16	2
2-4	0.92	0.99	0.53	0.09	3
2-5	0.995	0.997	0.11	0.11	1
2-5-1	0.99	0.998	0.2	0.13	2
2-5-3	0.991	1	0.14	0	1
2-5-4	0.96	0.999	0.35	0.09	3
2-5-3-1	0.99	0.998	0.2	0.1	1
2-5-3-4	0.99	0.993	0.23	0.11	2
2-5-3-1-4	0.988	0.994	0.32	0.17	1

The numbers 1 to 5 refers to input variables identified in Table 4.

## Conclusions

The current research was an effort to evaluate performance of an SMBR treating combined municipal and industrial wastewater compared with treating municipal and industrial wastewaters. The combined municipal and industrial wastewater showed more satisfactory treating features for wastewater treatment by an SMBR compared with the municipal and industrial wastewaters. Although the concentration of components in combined wastewater were almost half of the industrial wastewater, required HRT for combined wastewater was 7 h in comparison to 17 h for industrial wastewater. It was observed that treatment performance of the SMBR improves and HRT decreases noticeably by decreasing BOD and COD concentration to lower than 700 mg/L and 1000 mg/L, respectively. The results indicated that the combined wastewater improves treatment performance by increasing TBOD/TP ratio and setting BOD/COD ratio to 0.5. Therefore, effluent TP concentration was lower than 2 mg/L by increasing TBOD/TP ratio to more than 20. This study showed that it is possible to achieve a cost efficient wastewater treatment by SMBR for the combined wastewater because required HRT was at the same range for BOD, COD and ${\mathrm{NH}}_4^{+}-\mathrm{N}$ compared with the municipal and industrial wastewaters.

Treatment process models are efficient tools to assure proper operation and better control of wastewater treatment systems. ANNs have been successfully used for monitoring, controlling, classification and simulation of experimental data. In this study, an RBFANN was utilized in order to simulate effluent quality parameters of the SMBR treating combined municipal and industrial wastewater. An RBFANN is the most commonly used neural network for pattern recognition problems, it is also widely used for fault diagnosis and solving ill-conditioned problems. The RBFANN has the advantages of a fast learning process, a learning stage without any iteration of updating weights, robust ability, and adaptation capability compared with other ANNs such as MLP, RNN and ESN. The results showed that the training and testing procedure by RBFANN for effluent BOD, COD, ${\mathrm{NH}}_4^{+}-\mathrm{N}$ and TP were successful. The train and test models showed an almost perfect match between the experimental and predicted values of effluent BOD, COD, ${\mathrm{NH}}_4^{+}-\mathrm{N}$ and TP.

## Abbreviations

MBR: Membrane bioreactor
SMBR: Submerged membrane bioreactor
HRT: Hydraulic retention time
COD: Chemical oxygen demand
TN: Total nitrogen
BOD: Biochemical oxygen demand
TBOD/TP: Total BOD to total phosphorous
ASP: Activated sludge process
ASM: Activated sludge model
ANN: Artificial neural network
RBF: Radial basis function
MLP: Multi-layer perceptron
RNN: Recurrent neural network
ESN: Echo-state network
RBFANN: Radial basis function artificial neural network
MLVSS: Mixed liquor volatile suspended solids
TDS: Total dissolved solids
TSS: Total suspended solids
DO: Dissolved oxygen
MLSS: Mixed liquor suspended solids
MLPANN: Multi-layer perceptron artificial neural network
RMSE: Root mean squared error
R^2^: Coefficient of determination
K_s_: Half saturation coefficient
k: Maximum substrate utilization rate.K_d_, Endogenous decay coefficient
CBOD: Carbonaceous biochemical oxygen demand.
